# New Theory of Respiration and Circulation; Interesting Experiment on an Alligator

**Published:** 1852-03

**Authors:** 


ARTICLE V.

*New Theory of Resp'ration and Circulation---Interesting Experiment on an Alligator.*

[No apology is needed for laying the following correspondence before our readers. They will find it possessed of much interest, whatever view they may take of the theory attempted to be established. It will be seen that converts to it have risen up in no obscure place and of no mean standing----and Mrs. Williard may well be proud of the character and talent which have come to her aid in support of her new doctrines. It is proper to mention that she would gladly have withheld from publication the first part of Dr. Cartwright's letter—her "apotheosis" ; but its omission would have injured his graphic description, into which it is so ingeniously wrought, and it is therefore inserted. The theory in question—viz: that the motive powers of blood are in the lungs and not in the heart, is applicable, if true, to many useful purposes connected with both the healthy and diseased states of the human system ; and we now have on hand, for publication in the Journal, some extended remarks on this point by Dr. Cartwright.---Editor of Boston Med. & Surg. Jour.]

*New Orleans, Dec. 1st.*, 1851.

*Mrs. Williard.*

My Dear Madam :----I am about to write you a strange, original, but I hope not unwelcome letter, on a most interesting and important subject, which, judging from your writings, seems very *strangely* to have enlisted the energies of your whole soul. Believe me, that what I have to say is the literal truth, which I can prove in any court of justice, although in communicating it I shall be compelled to borrow a little from the language of fiction, to tell of truths more strange. I therefore beg leave to inform you, that I was present at what might, in mythological language, be termed your apotheosis, and that New Orleans is entitled to the credit of being the first to award the honor. In ancient times, on such occasions, a vast pile of faggots and aromatics was set on fire, and an eagle let loose from a high pinnacle to mount into the sky as the messenger of the mandate to defy a mortal. Thus in Rome of old. But in New Orleans, instead of kindling the fire in a pile of faggots, it was kindled by means of a blow-pipe, in the lungs of a dead leviathan of the Mississippi----or, in plain language, a saurian, crockodile or alligator, which *it brought to life!!* In its resuscitation your theory of "*the motive powers of the circulation of the blood*" was established beyond all doubt or dispute. The crockodile, an Egyptian divinity, ruscitated, instead of the eagle of imperial Rome let loose, was made the messenger of the mandate for your enrolment among the immortals. Many of the persons present on the occasion are not unknown to fame. Prof. C. G. Forshey, a learned chemist and distinguished topographical engineer in the United States' service, and whose essays on the hydrography of the Mississippi you have probably seen, superintended the inflating process. Dr. Bennett Dowler, destined to live in futurity as the discoverer of post-mortem muscular motion under percussion, and whose pathological investigations, although not half told, have already given him world-wide celebrity among the learned, performed the dissection of the throax of the dead. "*Niliaca fera*," literally laying the bosom bare by removing every covering that concealed the heart and lungs, thus enabling all to see what physiological phenomenon occurred in bringing the dead to life.

The alligator had been killed by tying the trachea. After it had been, to all appearances, perfectly dead for about an hour, it was brought up, from its cage on the ground, in a back lot, by some negroes, into the third story of a house on Tchoupitoulas street, the most public street in the city, and placed on the dissecting table. Dr. Dowler then dissected the throax, exposing the heart and lungs, and extended the dissection into the abdomen, so as to bring into view the organs of that region. The blood was all wiped away, and the vicera of the abdomen and throax exposed naked to the eye. Not a motion or sign of life occurred. I took the heart in my hand. It was dead and cold. A hole was afterwards cut in the trachea, below the ligature, and a blow-pipe introduced, which Prof. Forshey worked. Long and lustily did the Professor blow, the sweat streaming from every pore from the exertion, and no motion or sign of life appeared. The operation was about to be abandoned, when I (having full faith in the main conclusion of your theory, although I believe that some of the links of the ratiocination leading to it are defective) advised the inflation to be persevered in---and soon a faint quivering of moving blood in the diaphanous veins of the lungs began to be seen. The inflating process was continued with renewed energy, and at length the blood began to run in a stream from the lungs into the quiescent heart. Then the heart began to quiver, and soon to pulsate, and ere long signs of life began elsewhere to occur. The inflation being continued, the animal began to move. Dr. Powell undertook to hold it, and, although a strong muscular man, 'Caiman' became too strong for him and other assistants to hold. The inflation was stopped, and the saurian was bound with cords. The process was resumed, and had not been continued long, before Leviathan was himself again, and broke the cords as easily as did the strong man those of the Philistines. Becoming dangerous to the by-standers, and proving his title clear to his old epithets, "*formidabilis---immanis---terrificus---horrendus*"---the inflation was suspended, and the Sampson of the Mississippi was shorn of his strength, and fast bound to the table by strong ropes. Again the vital air was sent to his lungs, and again "*la grande Dragonne*," as the French call him, made the most vigorous exertions to get loose --biting and snapping at everything. The vivisection clearly proved, that the *primum mobile* of the circulation and the chief motive powers of the blood are in the lungs, and not in the heart. Dr. Dowler, having never read your work on the circulation, when he saw the blood in motion in the lungs before any movement occurred in the heart, supposed that atmospheric air endowed the globules of the blood with a self-locomotory power. But why seek for a hypothetical self-locomotory power in the globules themselves, when the laws of chemistry declare to us the development of a most active locomotory power in the caloric evolved in the transmission of venous into arterial blood? the capacity for heat, between the two, being so different, that the latter could contain insensible caloric enough to give it motion, although its sensible temperature were actually less than the former. Thus either moves nnder a sensible temperature below that required to move water.

The alligator is a good type of those animals called cold-blooded. Some learned doctors have enlisted the cold-blooded animals and sent them against your theory of the circulation, to batter it down and to cover it with ridicule. How surprised will they be to find that these very animals sent against it to demolish it, have built for it an imperishable rampart, against all assaults.

In regard to the hot-blooded, I have a very pretty gold snuff box, for which I am primarily indebted to faith in your theory, and secondarily to a lady, the mother of a child supposed to be dead, whose lungs I continued perseveringly to inflate until the *nouveau nè* came to life, and is still living and flourishing.

But I must reserve some remarks I have to make on the utility of your doctrines, reduced to practice, for another communication.

You will be surprised and pleased at seeing an intimate connection traced between red, healthy blood, and education, physical, moral and intellectual, and the great advantages pointed out, which your discovery, showing how it can be made at will, gives to the physician in the prevention and cure of a multiplicity of diseases and infirmaties, particularly some of those common, and most of those peculiar, to your sex; as also the hidden power, of which it is the spring, requiring only to be put in motion and properly regulated and assisted by other expedients of science, to confer on them, not only health, intellectual and moral superiority, but grace and beauty.

I have the honor to be, witn great respect,

Your ob't servant,

SAMUEL A. CARTWRIGHT, M. D.

---

*Troy*, *Dec.* 11, 1851.

*Dr. Cartwright.*

DEAR SIR :---Day before yesterday I received the wonderful account of the great "saurian" experiment by yourself and other eminent physiologists ; and I received it as, some years ago, you told Mr. and Mrs. Prewett, then of Natchez, on returning to them my work on the "Circulation," that I had made the announcement of my theory. "She has found it," you said, "she has found it; it is true!---but she has told it like a woman." The statement of your wonderful operations on the monster of the Mississippi, learned and scientific---unexceptionable—all but a little poetic *heathenry* in the introduction, of itself jocular and amusing—yet moved me to tears and to prayers. Now God be magnified, I said, I shall no longer be looked upon as an impudent pretender, shunned and even hated by some of those whose good opinion is most valuable, pitied by others, as subject to a species of monomania of my own sex, materially hindered. For the great interest and object of my life has been, and is, their improvement. This physiological subject, and history, have been made the two main episodes; and both have furnished examples of the general manner in which my mind, whether I will or no, must operate.

The various steps of my *publishing* this history, have resulted from the strong impulses of a religious duty; for it was felt by me to be a masculine theory, and its reputation rather dreaded than coverted, though a sense of justice, and perhaps a love of fame, would not allow me to permit another to claim it. Many efforts were fruitlessly made to get it before the public otherwise than publishing it in my own name. My small volume on "The Motive Powers which produce the circulation of the Blood," was drawn forth by the feeling that the long journey I was about to take, in which I visited your mighty river, whose exhilarating waters once drank, men grow so fearless, that they no longer care for death, either as respects themselves or their neighbors—being about to take this journey I felt it to be my duty to publish this theory *then*, as I might have no hereafter in which to do it.

Yet, the theory required to be received by the medical faculty before it could be said to be adopted. But where were the brave physicians who would dare—a woman having first promulgated it—to assert its truth and its importance? They ought to have been found among those who drink the courage giving waters of the Mississippi, and whose hearts partake of that generous chivalry in the service of grateful woman, which my adventures would show, is indigenous upon its banks. And you, Sir, who pronounced my "Eureka," guided alone by your clear perception of truth, you ought to have been in this affair as you are, "the man of destiny;" and *Leviathan*, breaking his bonds, is a fit emblem of what you have done.

All clear revelation must be "*from* faith *to* faith." In reading your letter, some persons present, not previously initiated, wore countenances, as I was astonished to perceive, of indignant incredulity; and on bringing them to explanation, I found that they regarded it as an unmitigated hoax!! and thought that Ferdinand Mendez Pinto was but a type of the author, whoever he might be. I however showed them so many evidences of its genuineness, especially the ability and learning, medical and literary, with which it was written, that they finally concluded that a man who could compose thus, would not stultify himself by a contemptible artifice. Nevetheless, in the announcement to the medical world of one of the most important and remarkable experiments upon their records, would it not be well to request Professor Forshey and Dr. Dowler, one or both, to give, in their own language, a statement to follow yours; so that by "two or three witnesses" the mouth of unbelief itself may be stopped.

In the mean time I will communicate with Dr. Smith, the editor of the Boston Medical Journal, and after copying your letter, send him the original; and perhaps as the duty of forwarding for publication your mythological exultation of myself, falls to me, I must send my answer too; to show that though I thank you from my heart, it is not so much that you offer me a robe of honor, as that you take a fool's cap from my head, and a heavy weight from my feeble shoulders; and chiefly, that I may now hope and expect, that the truth, which for nineteen years I have, by God's help, nursed in solitude and sickly shade, is from henceforth to emerge into free air, and vigorous sunlight, and to be a blessing to mankind.

With profound respect, I am, Sir,

Your friend and servant,

EMMA WILLARD.

